# Supplementary material for: Rugby Health and Well-Being Study: protocol for a UK-wide survey with health data cross-validation
Source: BMJ Open. 2021 Jan 29;11(1):e041037. doi: 10.1136/bmjopen-2020-041037 (PMC7849884; doi:10.1136/bmjopen-2020-041037)
Supplement: Author's manuscript [file bmjopen-2020-041037.draft_revisions.pdf]

BMJ Open is committed to open peer review. As part of this commitment we make the peer review history of every article we publish publicly available.

When an article is published we post the peer reviewers' comments and the authors' responses online. We also post the versions of the paper that were used during peer review. These are the versions that the peer review comments apply to.

The versions of the paper that follow are the versions that were submitted during the peer review process. They are not the versions of record or the final published versions. They should not be cited or distributed as the published version of this manuscript.

BMJ Open is an open access journal and the full, final, typeset and author-corrected version of record of the manuscript is available on our site with no access controls, subscription charges or pay-per-view fees (http://bmjopen.bmj.com).

If you have any questions on BMJ Open's open peer review process please email info.bmjopen@bmj.com

BMJ Open

The Rugby Health and Wellbeing Study: Protocol for a UK-wide Survey with Health Data Cross-validation

Journal:	*BMJ Open*
Manuscript ID	bmjopen-2020-041037
Article Type:	Protocol
Date Submitted by the Author:	29-May-2020

Complete List of Authors:	Panagodage Perera, Nirmala; University of Oxford, Nuffield Department of Orthopaedics, Rheumatology and Musculoskeletal Sciences; University of Oxford, Centre for Sport, Exercise and Osteoarthritis Research Versus Arthritis<br>Radojčić, Maja; University of Oxford, Nuffield Department of Orthopaedics, Rheumatology and Musculoskeletal Sciences ; University of Oxford, Centre for Sport, Exercise and Osteoarthritis Research Versus Arthritis<br>Filbay, Stephanie; University of Oxford, Nuffield Department of Orthopaedics, Rheumatology and Musculoskeletal Sciences; University of Oxford, Centre for Sport, Exercise and Osteoarthritis Research Versus Arthritis<br>Griffin, Steffan; Rugby Football Union; University of Edinburgh , Centre for Sport and Exercise<br>Gates, Lucy; University of Oxford, Nuffield Department of Orthopaedics, Rheumatology and Musculoskeletal Sciences; University of Southampton, Centre for Sport, Exercise and Osteoarthritis Research Versus Arthritis<br>Murray, Andrew; University of Edinburgh , Centre for Sport and Exercise; Scottish Rugby Union<br>Hawkes, Roger; British Association of Sport and Exercise Medicine<br>Arden, Nigel; University of Oxford, Nuffield Department of Orthopaedics, Rheumatology and Musculoskeletal Sciences; University of Oxford, Centre for Sport, Exercise and Osteoarthritis Research Versus Arthritis

Keywords:	SPORTS MEDICINE, PUBLIC HEALTH, EPIDEMIOLOGY

SCHOLARONE™
Manuscripts

I, the Submitting Author has the right to grant and does grant on behalf of all authors of the Work (as defined in the below author licence), an exclusive licence and/or a non-exclusive licence for contributions from authors who are: i) UK Crown employees; ii) where BMJ has agreed a CC-BY licence shall apply, and/or iii) in accordance with the terms applicable for US Federal Government officers or employees acting as part of their official duties; on a worldwide, perpetual, irrevocable, royalty-free basis to BMJ Publishing Group Ltd ("BMJ") its licensees and where the relevant Journal is co-owned by BMJ to the co-owners of the Journal, to publish the Work in this journal and any other BMJ products and to exploit all rights, as set out in our licence.

The Submitting Author accepts and understands that any supply made under these terms is made by BMJ to the Submitting Author unless you are acting as an employee on behalf of your employer or a postgraduate student of an affiliated institution which is paying any applicable article publishing charge ("APC") for Open Access articles. Where the Submitting Author wishes to make the Work available on an Open Access basis (and intends to pay the relevant APC), the terms of reuse of such Open Access shall be governed by a Creative Commons licence – details of these licences and which Creative Commons licence will apply to this Work are set out in our licence referred to above.

Other than as permitted in any relevant BMJ Author's Self Archiving Policies, I confirm this Work has not been accepted for publication elsewhere, is not being considered for publication elsewhere and does not duplicate material already published. I confirm all authors consent to publication of this Work and authorise the granting of this licence.

For peer review only

THE RUGBY HEALTH AND WELLBEING STUDY: PROTOCOL FOR A UK-WIDE SURVEY WITH HEALTH DATA CROSS-VALIDATION

Nirmala Kanthi Panagodage Perera[1,2,3,4], Maja R Radojčić[1,2,*], Stephanie R Filbay[1,2], Steffan A Griffin[5,6], Lucy Gates[1,7], Andrew Murray[6,8], Roger Hawkes [9], Nigel K Arden[1,2]

[1] Nuffield Department of Orthopaedics, Rheumatology and Musculoskeletal Sciences, University of Oxford, Oxford, United Kingdom

[2] Centre for Sport, Exercise and Osteoarthritis Research Versus Arthritis, University of Oxford, Oxford, United Kingdom

[3] Department of Health, Medicine and Caring Sciences, Linköping University, Linköping, Sweden

[4] School of Allied Health, Human Services and Sport, Latrobe University, Melbourne, Australia

[5] Rugby Football Union, Twickenham, United Kingdom

[6] Centre for Sport and Exercise, University of Edinburgh, Edinburgh, United Kingdom

[7] Centre for Sport, Exercise and Osteoarthritis Research Versus Arthritis, University of Southampton, Southampton, United Kingdom

[8] Scottish Rugby Union, Murrayfield, United Kingdom

[9] British Association of Sport and Exercise Medicine, Doncaster, United Kingdom

Correspondence:

[*] Maja R Radojčić, maja.radojcic@ndorms.ox.ac.uk, *The Botnar Research Centre, Nuffield Department of Orthopaedics, Rheumatology and Musculoskeletal Sciences, University of Oxford, Windmill Road, OX3 7LD, Oxford, United Kingdom*

For peer review only

ABSTRACT

Introduction Rugby football (Union and League) provide physical activity (PA) with related physical and mental health benefits. However, as a collision sport, rugby research and media coverage mostly focus on injuries in elite players while the overall impact on health and wellbeing remains unclear. This study aims to provide a greater understanding of the risks and benefits of rugby participation in a diverse sample of men and women, current and former rugby Union and League players from recreational to the elite level of play. We will explore: 1) joint-specific injuries and concussion; 2) joint pain and osteoarthritis; 3) medical and mental health conditions; 4) PA and sedentary behaviour; 5) wellbeing (quality of life, flourishing and resilience).

Methods and analysis The Rugby Health and Wellbeing Study is designed in two phases; (1) a UK-wide cross-sectional survey, and (2) cross-validation using health register data from Scotland. Participants will be at least 16 years old, current or former rugby players who have played rugby for at least one season. We will report standardised, level of play-, sex- and age-stratified prevalence of joint injury, concussion, medical conditions and PA. We will describe injury/concussion prevention expectations and protective equipment use. Rugby-related factors associated with injury, pain, osteoarthritis, PA, health and wellbeing will be explored in regression models. We will compare joint pain intensity and duration, elements of pain perception and wellbeing between recreational and elite players and further investigate these associations in regression models while controlling for confounding. In the second phase, we will validate self-reported with health register data, and provide further information on health care use.

Ethics and dissemination The Yorkshire and the Humber – Leeds East Research Ethics Committee (REC reference: 19/HY/0377) has approved this study (IRAS project ID 269424). The results will be disseminated through scientific publications, conferences and social media.

For peer review only

ARTICLE SUMMARY

Strengths and limitations of this study

- A large nationwide sample of current and former rugby participants of different playing standards and ages;
- A comprehensive data collection about injury experience and pain perception, physical activity and quality of life which will contribute to evidence-informed strategies to promote player welfare.
- The study will inform recommendations for the design of future prospective studies on health and wellbeing in current and former rugby players.
- Limitations include cross-sectional design, the potential for recall bias of self-reported data, and unavailability of health register data for all participants for the validation phase.

For peer review only

Introduction

Health is "a state of complete physical, mental and social well-being and not merely the absence of disease or infirmity"[1] as defined by the World Health Organisation. Growing evidence suggests that regular physical activity (PA) can provide longevity, physical and mental health benefits regardless of age, sex, geographical location and socioeconomic backgrounds, and provide economic benefits for communities[2-4] and improve wellbeing.[5 6] According to the Compendium of Physical Activities criteria,[7] rugby football (referred to as rugby herein) can be a source of moderate-to-vigorous PA.[8 9] As a collision sport, there are risks of injury and concussion[10-14] with acute injuries being the focus of the majority of research[15] and media coverage. Sports injuries are associated with adverse long-term health outcomes like increased risk of post-traumatic osteoarthritis (OA)[16 17] and joint replacement,[18] neurocognitive deficits[19] and decreased neuropsychological performance[20] and impaired quality of life.[16 21-23] However, on top of the physical benefits of sport participation,[24-26] psychosocial benefits include resilience, self-efficacy, enhanced coping, positivity and sense of belonging.[27-30] The overview of sport participation and medium/long-term health outcomes has been synthesised in football, tennis, golf[26] and cycling[31] but not in rugby.[15] Thus, there is a pressing need to evaluate the health and wellbeing benefits, as well as the risks involved in playing rugby.

The priority of global rugby governing bodies is player welfare and making rugby safe and enjoyable for all. Boys and girls, men and women throughout the world play rugby in two distinct codes, Union and League. There are many similarities between the codes and distinct differences like player morphology, tackle technique, a demographic and socioeconomic background that can influence overall benefit-risk relation. The rugby-related risk in different populations can be prevented by strategies that deal not only with prevention of injury occurrence but also with slowing disease development and reducing disability. The first step toward developing different prevention strategies is to define the magnitude, scope and characteristics of the problem through the systematic collection of data.[32]

For peer review only

Thus, the Rugby Health and Wellbeing Study aims to provide a comprehensive overview and a greater understanding of the potential risks and benefits of rugby participation. We will include current and former, recreational and elite rugby Union and League players, men and women from a variety of cultural backgrounds in the United Kingdom (UK) to explore (1) joint-specific injuries and concussion, (2) joint pain and OA, (3) medical and mental health conditions, (3) physical activity and sedentary behaviour, and (5) wellbeing, i.e. quality of life, flourishing and resilience.

Methods and analysis

Study design and participants

The Rugby Health and Wellbeing Study is designed as a cross-sectional study in two phases: phase 1 – an online survey, and phase 2 – data cross-validation from health register (Figure 1). The study is informed by the Cricket Health and Wellbeing Study. That study performed qualitative research work, i.e. interviews and patient and public involvement within the Centre for Sport, Exercise and Osteoarthritis Research Versus Arthritis, in the process of defining the content and wording of questions.[33 34] The present study incorporates the valuable feedback on the Cricket Health and Wellbeing Study, and also adds several additional sections of interest, cross-validation phase and adaptations specific to rugby.

Inclusion criteria for this study are people aged 16 years or older, who are current or former rugby players having played rugby for at least one season. We aim to recruit a diverse sample including recreational players, women and youth from a variety of cultural backgrounds as these groups are under-represented in existing rugby research.

Sample size

The study sample size is calculated using the equation for surveys,

$$Sample\ size = \frac{\frac{z^2 \times p(1-p)}{e^2}}{1 + \frac{z^2 \times p(1-p)}{e^2 N}}$$

For peer review only

where N – population size, p – proportion (standard for surveys 50%), e – margin of error (5%), and z – z-score (1.96 for 95% confidence).

According to the equation and the given constants, the sample size depends only on the population size (N). In 2016, there were 3,200,000 registered men and 27,500 women playing rugby in England.[35] For these populations, the required sample size is 385 men and 379 women. Given this slight difference, we assume equal-size groups, meaning that we need to recruit 770 current players for England. We expect similar numbers of former and current players to complete the survey such that we will need to recruit a total of 1,540 players. To optimise the study to be representative of the UK as a whole, we aim to recruit similar numbers of players for Scotland, Wales and Northern Ireland, leading to an overall sample size of 6,160 players. Finally, based on a response rate (10%) in the Cricket Health and Wellbeing Study,[36] we need to contact 61,600 current and former players to achieve the intended sample size.

Recruitment

We will employ several recruitment strategies to reach the intended sample size and diversity. Firstly, to recruit current rugby players, we will send emails to clubs registered with stakeholders asking to forward the recruitment letters to their players. Also, research team members will attend local clubs to provide further information and encourage participation. To reach former players as well, we will contact alumni organisations with established links to rugby in a similar manner asking to forward recruitment letters to their members. Further, in previous studies in sports conducted by our group, participants have been asked and consented to permission for future contact. We will contact these people by email. Additionally, there are online tools available (www.callforparticipant.com) where people have registered to participate in research studies. We will post the project details on this website to alert about our study. In all occasions, the email will provide information about the study, the Participant Information Sheet and a link to the survey.

Additionally, we will use standard advertisements and social media to reach a wider population. We will provide information about the study and ongoing recruitment through

For peer review only

advertisements in newspapers, magazines, flyers, posters, information sheets, notices, internet postings, public engagement activities by our group and traditional media appearances such as radio. Additionally, a press release will be issued through Centre for Sport, Exercise and Osteoarthritis Research Versus Arthritis to alert the public about the study and to recruit participants. Furthermore, the research team will use social media platforms such as Twitter to share the Participant Information Sheet and a link to the survey. The study will also be promoted via podcasts, blog posts, and newsletters.

Phase 1

All participants will complete the online survey – Phase 1. The survey link is specific per email address ensuring that each participant completes the survey only once. The online survey will be administered via electronic data capture software ALEA™ (FormsVision, Abcoude, the Netherlands) through the University of Southampton. It will start in mid-2020 and be active for six months. The survey completion requires a time commitment of 20-45 minutes owing to branching logic.

The survey is organised in several sections according to the study aims. Firstly, participants will report their demographic data such as age, height, weight, ethnicity, education level, and employment status, as well as smoking, recreational drug and alcohol use.

Joint-specific injury and concussion – We will collect data on playing status, rugby participation history, age and reason/s for retirement, joint injury and concussion history, protective equipment use, injury/concussion risk perception, prevention expectations and knowledge.[37 38]

Joint pain and osteoarthritis – Participants will report their current pain, as well as duration-based severe joint pain, i.e. pain on most days per month in the previous year. They will be asked to identify all affected joints including laterality on a predetermined list. The joint pain parameters of interest include intensity, chronicity, occurrence, and the effect on daily activities. Additionally, this part includes selected questions from the Pain-related Cognitive Processes Questionnaire (PCPQ)[39] where participants are asked to report cognitive,

For peer review only

behavioural and emotional aspects when experiencing pain. We will also collect data on the history of osteoarthritis and orthopaedic surgery.

Medical and mental health conditions – We will gather data about current and previous medical and mental health conditions diagnosed by a health professional,[16 40] stress, sleep and wellbeing, [38] effects of health problems on rugby participation[41 42] and access to health care.

Physical activity and sedentary behaviour – This section aims to collect data on types of other sports played, PA and sedentary behaviour over the past seven days using International Physical Activity Questionnaires – Short Form (IPAQ-SF) questionnaire[43] and perception of current PA levels.

Wellbeing – quality of life, flourishing and resilience – The last section of the survey focuses on the quality of life using the Short Form Health Survey 12 (SF-12),[44] enjoyment and physical/psychological benefits of rugby, resilience (European Social Survey),[45] and flourishing (Flourishing Scale).[46]

The comparison population-based cohorts

We will utilise data from two representative population-based cohorts in England as a comparison to calculate standardised morbidity ratios of medical conditions reported in our study. For this purpose, participants of the Rugby Health and Wellbeing Study will be age- and sex-matched to participants from the English Longitudinal Study of Ageing (ELSA) and the Health Survey for England (HSE), 2015 data.[47]

Phase 2

Phase 1 participants from Scotland who consent to share their full name and date of birth to enable matching with their Community Health Index (CHI) number will be included in Phase 2. This phase is aimed to validate self-reported health data from Phase 1 and gather additional information on health care use. It is restricted to Scottish residents due to the availability of electronic health register via electronic Data Research and Innovation Service (eDRIS) (https://www.isdscotland.org/Products-and-Services/EDRIS). Through eDRIS research support, prospectively collected comprehensive inpatient and outpatient electronic medical

For peer review only

records from 1981 to date is available for all Scottish residents. The aim of eDRIS is to support research use of administrative datasets by providing a single-entry point and end-to-end support to researchers. The high-quality eDRIS data provide national coverage across Scotland and the ability to link data to allow patient-based analysis. The International Classification of Diseases 9/10 codes are used in these electronic medical records to identify conditions resulting in hospitalisation. After matching survey participants to their CHI number, individual-level data on outpatient attendances (SMR00), admissions to general acute hospitals (SMR01), psychiatric hospitals (SMR04) and cancer registrations (SMR06) will be obtained. These will be used for providing agreement between health register data and Phase 1 medical and mental health data and reporting additional information if not included in Phase 1.

Statistical analysis

We will provide descriptive statistics for the participants in Phase 1 and Phase 2 and compare the rugby participants with a population-based comparison cohort for the result generalisation. We will report on the prevalence per 1,000 participants of self-reported joint-specific injuries, concussion, joint-specific pain, osteoarthritis and medical and mental health conditions. These will be standardised to the population but also stratified by age and playing position. Further, we will calculate standardised morbidity ratios (SMRs) for medical and mental health conditions, osteoarthritis, and joint replacement for rugby players when age- and sex-matched to participants from the population-based comparison cohort.

The explorative analyses will aim to determine the relationship between rugby-related factors and joint-specific injuries, joint-specific pain and osteoarthritis for current and former rugby players of all standards of play. This analysis will determine factors from training and playing history that are most likely to be associated with an increased risk of osteoarthritis. We will use multivariable regression models to assess the interactions and to control the associations for the influence of relevant confounding variables. Injury prevention expectations and risk perception will be compared between elite and recreational standards of play using a t-test or

For peer review only

a Mann–Whitney U test. We will describe protective equipment use. We will investigate pain parameters and elements of pain perception and compare these between recreational and elite players using a t-test. The relationship between elements of pain perception with pain intensity and duration while controlling for rugby-related factors will be assessed in linear and logistic regression models. Physical activity and sedentary behaviour in current and former rugby players will be analysed using descriptive statistics and stratified by age. Binary logistic regression analyses will be performed to determine the odds of meeting UK PA guidelines compared to a representative population-based comparison cohort and exploring factors associated with inactivity in former rugby players. The wellbeing of current and former rugby players will be explored using descriptive analyses. Factors related to quality of life, flourishing, and resilience will be determined in multivariable regression models.

In the validation phase, we will provide a percentage of agreement between survey and health register data and descriptive statistics on the health care use not captured by Phase 1.

Strengths and limitations

In this study, we will include a diverse nationwide sample of rugby participants – men and women, current and former rugby Union and League players from recreational to the elite level of play. It will be a comprehensive data collection about injury and pain experience, physical activity and quality of life that will contribute to evidence-informed strategies to promote player welfare and enhance the positive aspects of rugby. In a subsample, we will cross-validate self-reported data with electronic health records. Results of this study will provide recommendations for the design of future prospective studies.

Nevertheless, the study has some limitations. Firstly, it is a cross-sectional design with retrospective questions. We will investigate areas of interest in a single time point without considering time-dependent relations. However, this study will inform the design of future prospective rugby studies. Secondly, survey data are self-reported, and participants are asked about past events often subjected to recall bias. We have included several steps to minimise the recall bias. We ask about major injury i.e. injury leading to more than four weeks of reduced

For peer review only

participation in exercise, training or sport, the joint pain lasting most days per month, and medical and mental health conditions diagnosed by health professionals only. Further, the accuracy of retrospective self-reported injury compared to prospective reports has been shown in recreational Australian Football League players. Whereby, there was perfect agreement between a retrospective injury survey and prospective injury records over 12 months.[48] Also, 79% of the study participants accurately recalled the number of injuries, and 61% recalled the number, anatomical location and diagnoses of these injuries. Overall, the impact of recall bias is toward underreporting injuries and concussion.[48 49] Yet, underreporting other than recall bias can be an issue in current players. Our Participant Information Sheet includes a clarification that coaches/managers will not have access to the survey. Overall, the underreporting can potentially shift our results toward zero. Thirdly, our Phase 2 has some limitations, as well. While it is an objective way to validate self-reported health data and deal with underreporting, it is available for a subsample only. We cannot control the inclusion of the participants in the subsample, but we will report and discuss how the subsample represents the whole study sample. Also, we are limited by diagnostic codes and unable to explore all the health conditions. Finally, owing to the time commitment required for completing the survey, we prioritise the areas of interest over additional areas that potentially could confound some of the associations of interest, such as family history.

Patient and public involvement (PPI)

Centre for Sport, Exercise and Osteoarthritis Research Versus Arthritis has a PPI representative fully engaged in the research work providing suggestions and directions to explore, as well as feedback on the findings. We included in this study the PPI suggestions on diversity and interest in recreational players. In designing the survey, we involved men and women current and former rugby players and coaches, and international rugby representatives. They ensured that the survey is relevant and specific for rugby players to collect accurate and sport-specific self-reported data.

Ethics and dissemination

For peer review only

The Yorkshire and the Humber – Leeds East Research Ethics Committee (REC reference: 19/HY/0377) has approved the study (IRAS project ID 269424) in accordance to the Declaration of Helsinki of the World Medical Association. Written informed consent will be obtained from all participants. For young people aged 16 years or older, the UK law dictates that they can provide consent for medical treatment unless they are not able to make the decision. Following the same logic, individuals 16 years or older can consent to participate in this study.

We will use a multimodal approach for dissemination and knowledge translation. Following the publication policy of Versus Arthritis, results will be published in open-access peer-reviewed journals to enhance dissemination. In addition to scientific conference presentations, we will employ multimedia resources like infographics, animations, podcasts and blogs to subsequently disseminate and communicate results to the study participants, general public and stakeholders via social media platforms and mass media.

For peer review only

Authors' contributions

NKPP produced the first draft of the manuscript; MRR, SRF, SAG, LG, AM, RH and NKA provided critical comments on the manuscript. All authors were involved in designing the study and approved the final draft for submission.

Funding statement

This work was supported by Versus Arthritis grant number 21595 – Centre for Sport, Exercise and Osteoarthritis Research Versus Arthritis.

Competing interest statement

NKPP, MRR, SRF, LG, RH, NKA declare no competing interests. SAG works as a Sports Medicine Training Fellow for the Rugby Football Union and receives remuneration for clinical work in the sport. AM receives remuneration from Scottish Rugby Union for clinical work.

For peer review only

Figure legend

Figure 1 Study design and aims. eDRIS – electronic Data Research and Innovation Service.

For peer review only

References

1. World Health Organisation. Constitution Geneva, Swizerland: World Health Organisation,; 1948 [Available from: https://www.who.int/about/who-we-are/constitution accessed 6 December 2019.
2. Kohl 3rd HW, Craig CL, Lambert EV, et al. The pandemic of physical inactivity: global action for public health. *The lancet* 2012;380(9838):294-305.
3. Lee I-M, Shiroma EJ, Lobelo F, et al. Effect of physical inactivity on major non-communicable diseases worldwide: an analysis of burden of disease and life expectancy. *The lancet* 2012;380(9838):219-29.
4. Lim SS, Vos T, Flaxman AD, et al. A comparative risk assessment of burden of disease and injury attributable to 67 risk factors and risk factor clusters in 21 regions, 1990–2010: a systematic analysis for the Global Burden of Disease Study 2010. *The lancet* 2012;380(9859):2224-60.
5. Filbay SR, Pandya T, Thomas B, et al. Quality of life and life satisfaction in former athletes:: a systematic review and meta-analysis. *Sports Med* 2019
6. Penedo FJ, Dahn JR. Exercise and well-being: a review of mental and physical health benefits associated with physical activity. *Current opinion in psychiatry* 2005;18(2):189-93.
7. Ainsworth B, Haskell W, Herrmann S, et al. 2011 Compendium of physical activities: a second update of codes and MET values. *Med Sci Sports Exerc* 2011;43(8):1575-81.
8. Moy K, Scragg R, McLean G, et al. Metabolic equivalent (MET) intensities of culturally-specific physical activities performed by New Zealanders. *Special Series* 2006;5:6.
9. Smith DR, King RFGJ, Duckworth LC, et al. Energy expenditure of rugby players during a 14-day in-season period, measured using doubly labelled water. *Eur J Appl Physiol* 2018;118(3):647-56. doi: 10.1007/s00421-018-3804-4 [published Online First: 2018/01/20]
10. Gardner AJ, Iverson GL, Williams WH, et al. A systematic review and meta-analysis of concussion in rugby union. *Sports Med* 2014;44(12):1717-31.
11. Prien A, Grafe A, Rössler R, et al. Epidemiology of head injuries focusing on concussions in team contact sports: a systematic review. *Sports Med* 2018;48(4):953-69.
12. Freitag A, Kirkwood G, Scharer S, et al. Systematic review of rugby injuries in children and adolescents under 21 years. *Br J Sports Med* 2015;49(8):511-19.
13. Kirkwood G, Parekh N, Ofori-Asenso R, et al. Concussion in youth rugby union and rugby league: a systematic review. *Br J Sports Med* 2015;49(8):506-10.
14. Williams S, Trewartha G, Kemp S, et al. A meta-analysis of injuries in senior men's professional Rugby Union. *Sports medicine* 2013;43(10):1043-55.
15. Griffin SA, Panagodage Perera NK, Murray A, et al. The relationships between rugby union and health: a scoping review protocol. *BMJ Open Sport Exerc Med* 2019;5(1):e000593. doi: 10.1136/bmjsem-2019-000593 [published Online First: 2019/09/25]
16. Davies MA, Judge AD, Delmestri A, et al. Health amongst former rugby union players: A cross-sectional study of morbidity and health-related quality of life. *Sci Rep* 2017;7(1):11786.
17. Gouttebarge V, Kerkhoffs G, Lambert M. Prevalence and determinants of symptoms of common mental disorders in retired professional Rugby Union players. *European journal of sport science* 2016;16(5):595-602.
18. Ackerman IN, Bohensky MA, Kemp JL, et al. Quantifying the likelihood and costs of hip replacement surgery after sports injury: A population-level analysis. *Physical Therapy in Sport* 2020;41:9-15. doi: https://doi.org/10.1016/j.ptsp.2019.10.008
19. Hume P, Theadom A, Lewis G, et al. A comparison of cognitive function in former rugby union players compared with former non-contact-sport players and the impact of concussion history. *Sports Med* 2017;47(6):1209-20.
20. Cunningham J, Broglio S, Wilson F. Influence of playing rugby on long-term brain health following retirement: a systematic review and narrative synthesis. *BMJ Open Sport Exerc Med* 2018;4(1):e000356.
21. Houston MN, Hoch MC, Hoch JM. Health-related quality of life in athletes: a systematic review with Meta-analysis. *Journal of athletic training* 2016;51(6):442-53.
22. Guskiewicz KM, Marshall SW, Bailes J, et al. Recurrent concussion and risk of depression in retired professional football players. *Medicine and science in sports and exercise* 2007;39(6):903.
23. Didehbani N, Munro Cullum C, Mansinghani S, et al. Depressive symptoms and concussions in aging retired NFL players. *Archives of Clinical Neuropsychology* 2013;28(5):418-24.
24. Khan KM, Thompson AM, Blair SN, et al. Sport and exercise as contributors to the health of nations. *The Lancet* 2012;380(9836):59-64.

For peer review only

25. Lemez S, Baker J. Do elite athletes live longer? A systematic review of mortality and longevity in elite athletes. *Sports medicine-open* 2015;1(1):16.
26. Oja P, Titze S, Kokko S, et al. Health benefits of different sport disciplines for adults: systematic review of observational and intervention studies with meta-analysis. *Br J Sports Med* 2015;49(7):434-40.
27. Eime RM, Young JA, Harvey JT, et al. A systematic review of the psychological and social benefits of participation in sport for children and adolescents: informing development of a conceptual model of health through sport. *Int J Behav Nutr Phys Act* 2013;10(1):98. doi: 10.1186/1479-5868-10-98
28. Eime RM, Young JA, Harvey JT, et al. A systematic review of the psychological and social benefits of participation in sport for adults: informing development of a conceptual model of health through sport. *International journal of behavioral nutrition and physical activity* 2013;10(1):135.
29. Baker J, Fraser-Thomas J, Dionigi RA, et al. Sport participation and positive development in older persons. *European review of aging and physical activity* 2010;7(1):3.
30. Eime RM, Harvey JT, Brown WJ, et al. Does sports club participation contribute to health-related quality of life. *Med Sci Sports Exerc* 2010;42(5):1022-8.
31. Oja P, Titze S, Bauman A, et al. Health benefits of cycling: a systematic review. *Scandinavian journal of medicine & science in sports* 2011;21(4):496-509.
32. Finch C. A new framework for research leading to sports injury prevention. *J Sci Med Sport* 2006;9(1):3-9. doi: 10.1016/j.jsams.2006.02.009
33. Filbay SR, Bishop F, Peirce N, et al. Common attributes in retired professional cricketers that may enhance or hinder quality of life after retirement: a qualitative study. *BMJ Open* 2017;7(7):e016541. doi: 10.1136/bmjopen-2017-016541
34. Filbay SR, Bishop FL, Peirce N, et al. Physical activity in former elite cricketers and strategies for promoting physical activity after retirement from cricket: a qualitative study. *BMJ Open* 2017;7(11):e017785. doi: 10.1136/bmjopen-2017-017785
35. England Rugby. England Rugby's women and girls action plan. - RFU, 2017.
36. Cai H, Bullock GS, Sanchez-Santos MT, et al. Joint pain and osteoarthritis in former recreational and elite cricketers. *BMC Musculoskelet Disord* 2019;20(1):596. doi: 10.1186/s12891-019-2956-7 [published Online First: 2019/12/14]
37. McKay C, Merrett C, Emery C. Predictors of FIFA 11+ implementation intention in female adolescent soccer: An application of the Health Action Process Approach (HAPA) model. *International journal of environmental research and public health* 2016;13(7):657.
38. Perera NKP, Åkerlund I, Hägglund M. Motivation for sports participation, injury prevention expectations, injury risk perceptions and health problems in youth floorball players. *Knee Surgery, Sports Traumatology, Arthroscopy* 2019;27(11):3722-32. doi: 10.1007/s00167-019-05501-7
39. Day MA, Ward LC, Thorn BE, et al. The pain-related cognitive processes questionnaire: development and validation. *Pain Med* 2017;19(2):269-83.
40. Jones M, Davies M, Leyland K, et al. Osteoarthritis and other long-term health conditions in former elite cricketers. *J Sci Med Sport* 2018;21(6):558-63. doi: 10.1016/j.jsams.2017.10.013 [published Online First: 2017/11/02]
41. Clarsen B, Myklebust G, Bahr R. Development and validation of a new method for the registration of overuse injuries in sports injury epidemiology: the Oslo Sports Trauma Research Centre (OSTRC) overuse injury questionnaire. *Br J Sports Med* 2013;47(8):495-502.
42. Clarsen B, Rønsen O, Myklebust G, et al. The Oslo Sports Trauma Research Center questionnaire on health problems: a new approach to prospective monitoring of illness and injury in elite athletes. *Br J Sports Med* 2014;48(9):754-60.
43. Lee PH, Macfarlane DJ, Lam T, et al. Validity of the international physical activity questionnaire short form (IPAQ-SF): A systematic review. *Int J Behav Nutr Phys Act* 2011;8(1):115.
44. Wen J, Yk S, Yp L, et al. Quality of life, physical diseases, and psychological impairment among survivors 3 years after Wenchuan earthquake: a population based survey. *PLoS One* 2012;3
45. European Social Survey European Research Infrastructure Consortium (ESS ERIC). Source Questionnaire [Available from: https://www.europeansocialsurvey.org/methodology/ess_methodology/source_questionnaire/.
46. Diener E, Wirtz D, Tov W, et al. New well-being measures: Short scales to assess flourishing and positive and negative feelings. *Social Indicators Research* 2010;97(2):143-56.
47. NatCen Social Research UCL, Department of Epidemiology and Public Health. Health Survey for England, 2015. [data collection]. UK Data Service, 2017 [Accessed 25 May 2019]. Available from: http://doi.org/10.5255/UKDA-SN-8280-1.

48. Gabbe B, Finch C, Bennell K, et al. How valid is a self reported 12 month sports injury history? *British Journal of Sports Medicine* 2003;37(6):545-47.

49. Jenkins P, Earle-Richardson G, Slingerland DT, et al. Time dependent memory decay. *American Journal of Industrial Medicine* 2002;41(2):98-101. doi: 10.1002/ajim.10035

For peer review only

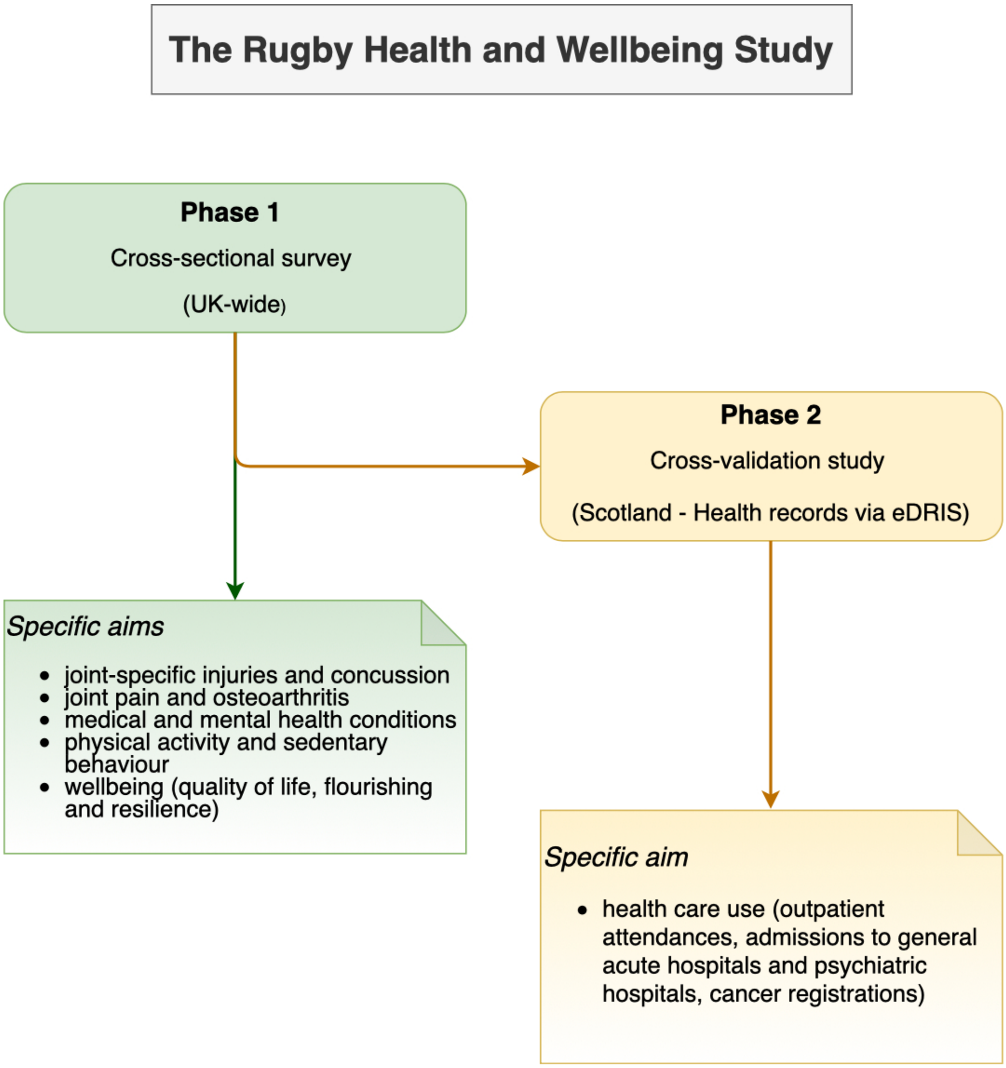

Figure 1 Study design and aims. eDRIS – electronic Data Research and Innovation Service.

90x96mm (300 x 300 DPI)

BMJ Open

The Rugby Health and Wellbeing Study: Protocol for a UK-wide Survey with Health Data Cross-validation

Journal:	*BMJ Open*
Manuscript ID	bmjopen-2020-041037.R1
Article Type:	Protocol
Date Submitted by the Author:	17-Dec-2020
Complete List of Authors:	Panagodage Perera, Nirmala; University of Oxford, Nuffield Department of Orthopaedics, Rheumatology and Musculoskeletal Sciences; University of Oxford, Centre for Sport, Exercise and Osteoarthritis Research Versus Arthritis<br>Radojčić, Maja; University of Oxford, Nuffield Department of Orthopaedics, Rheumatology and Musculoskeletal Sciences ; University of Oxford, Centre for Sport, Exercise and Osteoarthritis Research Versus Arthritis<br>Filbay, Stephanie; University of Oxford, Nuffield Department of Orthopaedics, Rheumatology and Musculoskeletal Sciences; University of Oxford, Centre for Sport, Exercise and Osteoarthritis Research Versus Arthritis<br>Griffin, Steffan; Rugby Football Union; University of Edinburgh , Centre for Sport and Exercise<br>Gates, Lucy; University of Oxford, Nuffield Department of Orthopaedics, Rheumatology and Musculoskeletal Sciences; University of Southampton, Centre for Sport, Exercise and Osteoarthritis Research Versus Arthritis<br>Murray, Andrew; University of Edinburgh , Centre for Sport and Exercise; Scottish Rugby Union<br>Hawkes, Roger; British Association of Sport and Exercise Medicine<br>Arden, Nigel; University of Oxford, Nuffield Department of Orthopaedics, Rheumatology and Musculoskeletal Sciences; University of Oxford, Centre for Sport, Exercise and Osteoarthritis Research Versus Arthritis
**Primary Subject Heading**:	Sports and exercise medicine
Secondary Subject Heading:	Epidemiology, Public health
Keywords:	SPORTS MEDICINE, PUBLIC HEALTH, EPIDEMIOLOGY

SCHOLARONE™
Manuscripts

I, the Submitting Author has the right to grant and does grant on behalf of all authors of the Work (as defined in the below author licence), an exclusive licence and/or a non-exclusive licence for contributions from authors who are: i) UK Crown employees; ii) where BMJ has agreed a CC-BY licence shall apply, and/or iii) in accordance with the terms applicable for US Federal Government officers or employees acting as part of their official duties; on a worldwide, perpetual, irrevocable, royalty-free basis to BMJ Publishing Group Ltd ("BMJ") its licensees and where the relevant Journal is co-owned by BMJ to the co-owners of the Journal, to publish the Work in this journal and any other BMJ products and to exploit all rights, as set out in our licence.

The Submitting Author accepts and understands that any supply made under these terms is made by BMJ to the Submitting Author unless you are acting as an employee on behalf of your employer or a postgraduate student of an affiliated institution which is paying any applicable article publishing charge ("APC") for Open Access articles. Where the Submitting Author wishes to make the Work available on an Open Access basis (and intends to pay the relevant APC), the terms of reuse of such Open Access shall be governed by a Creative Commons licence – details of these licences and which Creative Commons licence will apply to this Work are set out in our licence referred to above.

Other than as permitted in any relevant BMJ Author's Self Archiving Policies, I confirm this Work has not been accepted for publication elsewhere, is not being considered for publication elsewhere and does not duplicate material already published. I confirm all authors consent to publication of this Work and authorise the granting of this licence.

For peer review only

THE RUGBY HEALTH AND WELLBEING STUDY: PROTOCOL FOR A UK-WIDE SURVEY WITH HEALTH DATA CROSS-VALIDATION

Nirmala Kanthi Panagodage Perera[1,2,3,4], Maja R Radojčić[1,2,*], Stephanie R Filbay[1,2], Steffan A Griffin[5,6], Lucy Gates[1,7], Andrew Murray[6,8], Roger Hawkes [9], Nigel K Arden[1,2]

[1] Nuffield Department of Orthopaedics, Rheumatology and Musculoskeletal Sciences, University of Oxford, Oxford, United Kingdom

[2] Centre for Sport, Exercise and Osteoarthritis Research Versus Arthritis, University of Oxford, Oxford, United Kingdom

[3] Department of Health, Medicine and Caring Sciences, Linköping University, Linköping, Sweden

[4] School of Allied Health, Human Services and Sport, Latrobe University, Melbourne, Australia

[5] Rugby Football Union, Twickenham, United Kingdom

[6] Centre for Sport and Exercise, University of Edinburgh, Edinburgh, United Kingdom

[7] Centre for Sport, Exercise and Osteoarthritis Research Versus Arthritis, University of Southampton, Southampton, United Kingdom

[8] Scottish Rugby Union, Murrayfield, United Kingdom

[9] British Association of Sport and Exercise Medicine, Doncaster, United Kingdom

Correspondence:

[*] Maja R Radojčić, maja.radojcic@ndorms.ox.ac.uk, *The Botnar Research Centre, Nuffield Department of Orthopaedics, Rheumatology and Musculoskeletal Sciences, University of Oxford, Windmill Road, OX3 7LD, Oxford, United Kingdom*

For peer review only

ABSTRACT

Introduction Rugby football (Union and League) provide physical activity (PA) with related physical and mental health benefits. However, as a collision sport, rugby research and media coverage predominantly focus on injuries in elite players while the overall impact on health and wellbeing remains unclear. This study aims to provide a greater understanding of the risks and benefits of rugby participation in a diverse sample of men and women, current and former rugby Union and League players from recreational to the elite level of play. We will explore: 1) joint-specific injuries and concussion; 2) joint pain and osteoarthritis; 3) medical and mental health conditions; 4) PA and sedentary behaviour; 5) wellbeing (quality of life, flourishing and resilience).

Methods and analysis The Rugby Health and Wellbeing Study is designed in two phases; (1) a UK-wide cross-sectional survey, and (2) cross-validation using health register data from Scotland. Participants will be at least 16 years old, current or former rugby players who have played rugby for at least one season. We will report standardised, level of play-, sex- and age-stratified prevalence of joint injury, concussion, medical conditions and PA. We will describe injury/concussion prevention expectations and protective equipment use. Rugby-related factors associated with injury, pain, osteoarthritis, PA, health and wellbeing will be explored in regression models. We will compare joint pain intensity and duration, elements of pain perception and wellbeing between recreational and elite players and further investigate these associations in regression models while controlling for confounding variables. In the second phase, we will validate self-reported with health register data, and provide further information on health care use.

Ethics and dissemination The Yorkshire and the Humber – Leeds East Research Ethics Committee (REC reference: 19/HY/0377) has approved this study (IRAS project ID 269424). The results will be disseminated through scientific publications, conferences and social media.

For peer review only

ARTICLE SUMMARY

Strengths and limitations of this study

- A large nationwide sample of current and former rugby participants of different playing standards and ages;
- A comprehensive data collection about injury experience and pain perception, physical activity and quality of life which will contribute to evidence-informed strategies to promote player welfare.
- The study will inform recommendations for the design of future prospective studies on health and wellbeing in current and former rugby players.
- Limitations include cross-sectional design, the potential for recall bias of self-reported data, and unavailability of health register data for all participants for the validation phase.

For peer review only

Introduction

Health is "a state of complete physical, mental and social wellbeing and not merely the absence of disease or infirmity"[1] as defined by the World Health Organisation. Growing evidence suggests that regular physical activity (PA) can provide longevity, physical and mental health benefits regardless of age, sex, geographical location and socioeconomic backgrounds, and provide economic benefits for communities[2-4] and improve wellbeing.[5 6] According to the Compendium of Physical Activities criteria,[7] rugby football (referred to as rugby herein) can be a source of moderate-to-vigorous PA.[8 9] As a collision sport, there are risks of musculoskeletal injury and concussion[10-15] with acute injuries being the focus of the majority of research[16] and media coverage. The overall incidence of musculoskeletal injuries ranged from 19.6 for elite women to 62 and 148 injuries per 1,000 match-hours for amateur and elite men players, respectively.[17-19] Among these, injuries of the lower and upper limb joints, and neck have been the most commonly reported.[20] Additionally, the overall incidence of reported concussion ranged from 0.55 at professional women to 2.08 and 4.73 per 1,000 match-hours at amateur and professional men level, respectively.[10] These injuries have been associated with adverse long-term health outcomes like increased risk of chronic pain, post-traumatic osteoarthritis (OA)[21 22] and joint replacement,[23] neurocognitive deficits[24] and decreased neuropsychological performance[25] and impaired quality of life.[21 26-28] However, on top of the physical benefits of sport participation,[29-31] team-sport based psychosocial benefits include resilience, self-efficacy, enhanced coping, positivity and sense of belonging.[32-35] The overview of sport participation and medium/long-term health outcomes has been synthesised in football, tennis, golf[31] and cycling[36] but not in rugby.[16] Thus, there is a pressing need to evaluate the health and wellbeing benefits, as well as the risks involved in playing rugby.

The priority of global rugby governing bodies is player welfare and making rugby safe and enjoyable for all. Boys and girls, men and women throughout the world play rugby in two distinct codes, Union and League. There are many similarities between the codes and distinct differences like player morphology, tackle technique, a demographic and socioeconomic

For peer review only

background that can influence overall benefit-risk relation. The rugby-related risk in different populations can be prevented by strategies that deal not only with prevention of injury occurrence but also with slowing disease development and reducing disability. The first step toward developing different prevention strategies is to define the magnitude, scope and characteristics of the problem through the systematic collection of data.[37]

Thus, the Rugby Health and Wellbeing Study aims to provide a comprehensive overview and a greater understanding of the potential risks and benefits of rugby participation. We will include current and former, recreational and elite rugby Union and League players, men and women from a variety of cultural backgrounds in the United Kingdom (UK) to explore (1) joint-specific injuries and concussion, (2) joint pain and OA, (3) medical and mental health conditions, (3) physical activity and sedentary behaviour, and (5) wellbeing, i.e. quality of life, flourishing and resilience.

Methods and analysis

Study design and participants

The Rugby Health and Wellbeing Study is designed as a cross-sectional study in two phases: phase 1 – an online survey, and phase 2 – data cross-validation from health register (Figure 1). The study is informed by the Cricket Health and Wellbeing Study. That study performed qualitative research work, i.e. interviews and patient and public involvement within the Centre for Sport, Exercise and Osteoarthritis Research Versus Arthritis, in the process of defining the content and wording of questions.[38 39] The present study incorporates the valuable feedback on the Cricket Health and Wellbeing Study, and also adds several additional sections of interest, cross-validation phase and adaptations specific to rugby.

Inclusion criteria for this study are people aged 16 years or older, who are current or former rugby players having played rugby for at least one season. We aim to recruit a diverse sample including recreational players, women and youth from a variety of cultural backgrounds as these groups are under-represented in existing rugby research.

Sample size

For peer review only

The study sample size is calculated using the equation for surveys,[40]

$$Sample\ size = \frac{\frac{z^2 \times p(1-p)}{e^2}}{1 + \frac{z^2 \times p(1-p)}{e^2 N}}$$

where N – population size, p – proportion (standard for surveys 50%), e – margin of error (5%), and z – z-score (1.96 for 95% confidence).

According to the equation and the given constants, the sample size depends only on the population size (N). In 2016, there were 3,200,000 registered men and 27,500 women playing rugby in England.[41] For these populations, the required sample size is 385 men and 379 women. Given this slight difference, we assume equal-size groups, meaning that we need to recruit 770 current players for England. We expect similar numbers of former and current players to complete the survey such that we will need to recruit a total of 1,540 players. To optimise the study to be representative of the UK, we considered that the population size of England is 80% of the UK population and extrapolated the same percentage to rugby players. Thus, we will aim to recruit an overall sample size of 1,925 players across the UK. Finally, based on a response rate (10%) in the Cricket Health and Wellbeing Study,[42] we need to contact 19,250 current and former players to achieve the intended sample size.

Recruitment

We will employ several recruitment strategies to reach the intended sample size and diversity while ensuring stratified sampling per current and former players followed by simple random sampling. Firstly, to recruit current rugby players, we will send emails to clubs registered with stakeholders asking to forward the recruitment letters to their players. Also, research team members will attend local clubs to provide further information and encourage participation. To reach former players as well, we will contact alumni organisations with established links to rugby in a similar manner asking to forward recruitment letters to their members. Further, in previous studies in sports conducted by our group, participants have been asked and consented to permission for future contact. We will contact these people by email. Additionally,

For peer review only

there are online tools available (www.callforparticipant.com) where people have registered to participate in research studies. We will post the project details on this website to alert about our study. In all occasions, the email will provide information about the study, the Participant Information Sheet and a link to the survey.

Additionally, we will use standard advertisements and social media to reach a wider population. We will provide information about the study and ongoing recruitment through advertisements in newspapers, magazines, flyers, posters, information sheets, notices, internet postings, public engagement activities by our group and traditional media appearances such as radio. Additionally, a press release will be issued through Centre for Sport, Exercise and Osteoarthritis Research Versus Arthritis to alert the public about the study and to recruit participants. Furthermore, the research team will use social media platforms such as Twitter to share the Participant Information Sheet and a link to the survey. The study will also be promoted via podcasts, blog posts, and newsletters.

Phase 1

All participants will complete the online survey – Phase 1. The survey link is specific per email address ensuring that each participant completes the survey only once. The online survey will be administered via electronic data capture software ALEA™ (FormsVision, Abcoude, the Netherlands) through the University of Southampton. It will start in 2021 and be active for six months. The survey completion requires a time commitment of 20-45 minutes owing to branching logic.

The survey is organised in several sections according to the study aims. Firstly, participants will report their demographic data such as age, height, weight, ethnicity, education level, and employment status, as well as smoking, recreational drug and alcohol use.

Joint-specific injury and concussion – We will collect data on playing status, rugby participation history, age and reason/s for retirement, joint injury and concussion history, protective equipment use, injury/concussion risk perception, prevention expectations and knowledge.[43] [44]

For peer review only

Joint pain and osteoarthritis – Participants will report their current pain, as well as duration-based severe joint pain, i.e. pain on most days per month in the previous year. They will be asked to identify all affected joints including laterality on a predetermined list. The joint pain parameters of interest include intensity, chronicity, occurrence, and the effect on daily activities. Additionally, this part includes selected questions from the Pain-related Cognitive Processes Questionnaire (PCPQ)[45] where participants are asked to report cognitive, behavioural and emotional aspects when experiencing pain. We will also collect data on the history of osteoarthritis and orthopaedic surgery.

Medical and mental health conditions – We will gather data about current and previous medical and mental health conditions diagnosed by a health professional,[21 46] stress, sleep and wellbeing, [44] effects of health problems on rugby participation[47 48] and access to health care.

Physical activity and sedentary behaviour – This section aims to collect data on types of other sports played, PA and sedentary behaviour over the past seven days using International Physical Activity Questionnaires – Short Form (IPAQ-SF) questionnaire[49] and perception of current PA levels.

Wellbeing – quality of life, flourishing and resilience – The last section of the survey focuses on the quality of life using the Short Form Health Survey 12 (SF-12),[50] enjoyment and physical/psychological benefits of rugby, resilience (European Social Survey),[51] and flourishing (Flourishing Scale).[52]

The comparison population-based cohorts

We will utilise data from two representative population-based cohorts in England as a comparison to calculate standardised morbidity ratios of medical conditions reported in our study. For this purpose, participants of the Rugby Health and Wellbeing Study will be age- and sex-matched to participants from the English Longitudinal Study of Ageing (ELSA) and the Health Survey for England (HSE), 2015 data.[53]

Phase 2

For peer review only

Phase 1 participants from Scotland who consent to share their full name and date of birth to enable matching with their Community Health Index (CHI) number will be included in Phase 2. This phase is aimed to validate self-reported health data from Phase 1 and gather additional information on health care use. It is restricted to Scottish residents due to the availability of electronic health register via electronic Data Research and Innovation Service (eDRIS) (https://www.isdscotland.org/Products-and-Services/EDRIS). Through eDRIS research support, prospectively collected comprehensive inpatient and outpatient electronic medical records from 1981 to date is available for all Scottish residents. The aim of eDRIS is to support research use of administrative datasets by providing a single-entry point and end-to-end support to researchers. The high-quality eDRIS data provide national coverage across Scotland and the ability to link data to allow patient-based analysis. The International Classification of Diseases 9/10 codes are used in these electronic medical records to identify conditions resulting in hospitalisation. After matching survey participants to their CHI number, individual-level data on outpatient attendances (SMR00), admissions to general acute hospitals (SMR01), psychiatric hospitals (SMR04) and cancer registrations (SMR06) will be obtained. These will be used for providing agreement between health register data and Phase 1 medical and mental health data and reporting additional information if not included in Phase 1.

Statistical analysis

We will provide descriptive statistics for the participants in Phase 1 and Phase 2 and compare the rugby participants with a population-based comparison cohort for the result generalisation. We will report on the prevalence per 1,000 participants of self-reported joint-specific injuries, concussion, joint-specific pain, osteoarthritis and medical and mental health conditions. These will be standardised to the population but also stratified by age, sex and playing position. Further, we will calculate standardised morbidity ratios (SMRs) for medical and mental health conditions, osteoarthritis, and joint replacement for rugby players when age- and sex-matched to participants from the population-based comparison cohort.

For peer review only

The explorative analyses will aim to determine the relationship between rugby-related factors and joint-specific injuries, joint-specific pain and osteoarthritis for women and men, current and former rugby players of all standards of play. This analysis will determine factors from training and playing history that are most likely to be associated with an increased risk of osteoarthritis. We will use multivariable regression models to assess the interactions and to control the associations for the influence of relevant confounding variables. Injury prevention expectations and risk perception will be compared between elite and recreational standards of play using a t-test or a Mann–Whitney U test. We will describe protective equipment use. We will investigate pain parameters and elements of pain perception and compare these between recreational and elite players using a t-test. The relationship between elements of pain perception with pain intensity and duration while controlling for rugby-related factors will be assessed in linear and logistic regression models. Physical activity and sedentary behaviour in current and former rugby players will be analysed using descriptive statistics and stratified by age and sex. Binary logistic regression analyses will be performed to determine the odds of meeting UK PA guidelines compared to a representative population-based comparison cohort and exploring factors associated with inactivity in former rugby players. The wellbeing of current and former rugby players will be explored using descriptive analyses. Factors related to quality of life, flourishing, and resilience will be determined in multivariable regression models.

In the validation phase, we will provide a percentage of agreement between survey and health register data and descriptive statistics on the health care use not captured by Phase 1.

Strengths and limitations

In this study, we will include a diverse nationwide sample of rugby participants – men and women, current and former rugby Union and League players from recreational to the elite level of play. It will be a comprehensive data collection about injury and pain experience, physical activity and quality of life that will contribute to evidence-informed strategies to promote player welfare and enhance the positive aspects of rugby. In a subsample, we will cross-validate self-

For peer review only

reported data with electronic health records. Results of this study will provide recommendations for the design of future prospective studies.

Nevertheless, the study has some limitations. Firstly, it is a cross-sectional design with retrospective questions. We will investigate areas of interest in a single time point without considering time-dependent relations. However, this study will inform the design of future prospective rugby studies. Secondly, survey data are self-reported, and participants are asked about past events often subjected to recall bias. We have included several steps to minimise the recall bias. We ask about major injury i.e. injury leading to more than four weeks of reduced participation in exercise, training or sport, the joint pain lasting most days per month, and medical and mental health conditions diagnosed by health professionals only. Further, the accuracy of retrospective self-reported injury compared to prospective reports has been shown in recreational Australian Football League players. Whereby, there was perfect agreement between a retrospective injury survey and prospective injury records over 12 months.[54] Also, 79% of the study participants accurately recalled the number of injuries, and 61% recalled the number, anatomical location and diagnoses of these injuries. Overall, the impact of recall bias is toward underreporting injuries and concussion.[54 55] Yet, underreporting other than recall bias can be an issue in current players. Our Participant Information Sheet includes a clarification that coaches/managers will not have access to the survey. Overall, the underreporting can potentially shift our results toward zero. Thirdly, our Phase 2 has some limitations, as well. While it is an objective way to validate self-reported health data and deal with underreporting, it is available for a subsample only. We cannot control the inclusion of the participants in the subsample, but we will report and discuss how the subsample represents the whole study sample. Also, we are limited by diagnostic codes and unable to explore all the health conditions. Finally, owing to the time commitment required for completing the survey, we prioritise the areas of interest over additional areas that potentially could confound some of the associations of interest, such as family history.

Patient and public involvement (PPI)

For peer review only

Centre for Sport, Exercise and Osteoarthritis Research Versus Arthritis has a PPI representative fully engaged in the research work providing suggestions and directions to explore, as well as feedback on the findings. We included in this study the PPI suggestions on diversity and interest in recreational players. In designing the survey, we involved men and women current and former rugby players and coaches, and international rugby representatives. They ensured that the survey is relevant and specific for rugby players to collect accurate and sport-specific self-reported data.

Ethics and dissemination

The Yorkshire and the Humber – Leeds East Research Ethics Committee (REC reference: 19/HY/0377) has approved the study (IRAS project ID 269424) in accordance to the Declaration of Helsinki of the World Medical Association. Written informed consent will be obtained from all participants. For young people aged 16 years or older, the UK law dictates that they can provide consent for medical treatment unless they are not able to make the decision. Following the same logic, individuals 16 years or older can consent to participate in this study.

We will use a multimodal approach for dissemination and knowledge translation. Following the publication policy of Versus Arthritis, results will be published in open-access peer-reviewed journals to enhance dissemination. In addition to scientific conference presentations, we will employ multimedia resources like infographics, animations, podcasts and blogs to subsequently disseminate and communicate results to the study participants, general public and stakeholders via social media platforms and mass media.

For peer review only

Authors' contributions

NKPP produced the first draft of the manuscript; MRR, SRF, SAG, LG, AM, RH and NKA provided critical comments on the manuscript. All authors were involved in designing the study and approved the final draft for submission.

Funding statement

This work was supported by Versus Arthritis grant number 21595 – Centre for Sport, Exercise and Osteoarthritis Research Versus Arthritis.

Competing interest statement

NKPP, MRR, SRF, LG, RH, NKA declare no competing interests. SAG works as a Sports Medicine Training Fellow for the Rugby Football Union and receives remuneration for clinical work in the sport. AM receives remuneration from Scottish Rugby Union for clinical work.

For peer review only

Figure legend

Figure 1 Study design and aims. eDRIS – electronic Data Research and Innovation Service.

For peer review only

References

1. World Health Organisation. Constitution Geneva, Swizerland: World Health Organisation,; 1948 [Available from: https://www.who.int/about/who-we-are/constitution accessed 6 December 2019.
2. Kohl 3rd HW, Craig CL, Lambert EV, et al. The pandemic of physical inactivity: global action for public health. *The lancet* 2012;380(9838):294-305.
3. Lee I-M, Shiroma EJ, Lobelo F, et al. Effect of physical inactivity on major non-communicable diseases worldwide: an analysis of burden of disease and life expectancy. *The lancet* 2012;380(9838):219-29.
4. Lim SS, Vos T, Flaxman AD, et al. A comparative risk assessment of burden of disease and injury attributable to 67 risk factors and risk factor clusters in 21 regions, 1990–2010: a systematic analysis for the Global Burden of Disease Study 2010. *The lancet* 2012;380(9859):2224-60.
5. Filbay SR, Pandya T, Thomas B, et al. Quality of life and life satisfaction in former athletes:: a systematic review and meta-analysis. *Sports Med* 2019
6. Penedo FJ, Dahn JR. Exercise and well-being: a review of mental and physical health benefits associated with physical activity. *Current opinion in psychiatry* 2005;18(2):189-93.
7. Ainsworth B, Haskell W, Herrmann S, et al. 2011 Compendium of physical activities: a second update of codes and MET values. *Med Sci Sports Exerc* 2011;43(8):1575-81.
8. Moy K, Scragg R, McLean G, et al. Metabolic equivalent (MET) intensities of culturally-specific physical activities performed by New Zealanders. *Special Series* 2006;5:6.
9. Smith DR, King RFGJ, Duckworth LC, et al. Energy expenditure of rugby players during a 14-day in-season period, measured using doubly labelled water. *Eur J Appl Physiol* 2018;118(3):647-56. doi: 10.1007/s00421-018-3804-4 [published Online First: 2018/01/20]
10. Gardner AJ, Iverson GL, Williams WH, et al. A systematic review and meta-analysis of concussion in rugby union. *Sports Med* 2014;44(12):1717-31.
11. Prien A, Grafe A, Rössler R, et al. Epidemiology of head injuries focusing on concussions in team contact sports: a systematic review. *Sports Med* 2018;48(4):953-69.
12. Freitag A, Kirkwood G, Scharer S, et al. Systematic review of rugby injuries in children and adolescents under 21 years. *Br J Sports Med* 2015;49(8):511-19.
13. Kirkwood G, Parekh N, Ofori-Asenso R, et al. Concussion in youth rugby union and rugby league: a systematic review. *Br J Sports Med* 2015;49(8):506-10.
14. Williams S, Trewartha G, Kemp S, et al. A meta-analysis of injuries in senior men's professional Rugby Union. *Sports medicine* 2013;43(10):1043-55.
15. Griffin SA, Panagodage Perera NK, Murray A, et al. The relationships between rugby union, and health and well-being: a scoping review. *Br J Sports Med* 2020 doi: 10.1136/bjsports-2020-102085 [published Online First: 2020/10/30]
16. Griffin SA, Panagodage Perera NK, Murray A, et al. The relationships between rugby union and health: a scoping review protocol. *BMJ Open Sport Exerc Med* 2019;5(1):e000593. doi: 10.1136/bmjsem-2019-000593 [published Online First: 2019/09/25]
17. Abarca-Gómez L, Abdeen ZA, Hamid ZA, et al. Worldwide trends in body-mass index, underweight, overweight, and obesity from 1975 to 2016: a pooled analysis of 2416 population-based measurement studies in 128·9 million children, adolescents, and adults. *Lancet* 2017;390(10113):2627-42.
18. King D, Gissane C, Clark T, et al. The Incidence of Match and Training Injuries in Rugby League: A Pooled Data Analysis of Published Studies. *International Journal of Sports Science & Coaching* 2014;9(2):417-32. doi: 10.1260/1747-9541.9.2.417
19. King D, Hume P, Cummins C, et al. Match and Training Injuries in Women's Rugby Union: A Systematic Review of Published Studies. *Sports Med* 2019;49(10):1559-74. doi: 10.1007/s40279-019-01151-4 [published Online First: 2019/07/12]

For peer review only

20. Williams S, Trewartha G, Kemp S, et al. A meta-analysis of injuries in senior men's professional Rugby Union. *Sports Med* 2013;43(10):1043-55. doi: 10.1007/s40279-013-0078-1 [published Online First: 2013/07/11]
21. Davies MA, Judge AD, Delmestri A, et al. Health amongst former rugby union players: A cross-sectional study of morbidity and health-related quality of life. *Sci Rep* 2017;7(1):11786.
22. Gouttebarge V, Kerkhoffs G, Lambert M. Prevalence and determinants of symptoms of common mental disorders in retired professional Rugby Union players. *European journal of sport science* 2016;16(5):595-602.
23. Ackerman IN, Bohensky MA, Kemp JL, et al. Quantifying the likelihood and costs of hip replacement surgery after sports injury: A population-level analysis. *Physical Therapy in Sport* 2020;41:9-15. doi: https://doi.org/10.1016/j.ptsp.2019.10.008
24. Hume P, Theadom A, Lewis G, et al. A comparison of cognitive function in former rugby union players compared with former non-contact-sport players and the impact of concussion history. *Sports Med* 2017;47(6):1209-20.
25. Cunningham J, Broglio S, Wilson F. Influence of playing rugby on long-term brain health following retirement: a systematic review and narrative synthesis. *BMJ Open Sport Exerc Med* 2018;4(1):e000356.
26. Houston MN, Hoch MC, Hoch JM. Health-related quality of life in athletes: a systematic review with Meta-analysis. *Journal of athletic training* 2016;51(6):442-53.
27. Guskiewicz KM, Marshall SW, Bailes J, et al. Recurrent concussion and risk of depression in retired professional football players. *Medicine and science in sports and exercise* 2007;39(6):903.
28. Didehbani N, Munro Cullum C, Mansinghani S, et al. Depressive symptoms and concussions in aging retired NFL players. *Archives of Clinical Neuropsychology* 2013;28(5):418-24.
29. Khan KM, Thompson AM, Blair SN, et al. Sport and exercise as contributors to the health of nations. *The Lancet* 2012;380(9836):59-64.
30. Lemez S, Baker J. Do elite athletes live longer? A systematic review of mortality and longevity in elite athletes. *Sports medicine-open* 2015;1(1):16.
31. Oja P, Titze S, Kokko S, et al. Health benefits of different sport disciplines for adults: systematic review of observational and intervention studies with meta-analysis. *Br J Sports Med* 2015;49(7):434-40.
32. Eime RM, Young JA, Harvey JT, et al. A systematic review of the psychological and social benefits of participation in sport for children and adolescents: informing development of a conceptual model of health through sport. *Int J Behav Nutr Phys Act* 2013;10(1):98. doi: 10.1186/1479-5868-10-98
33. Eime RM, Young JA, Harvey JT, et al. A systematic review of the psychological and social benefits of participation in sport for adults: informing development of a conceptual model of health through sport. *International journal of behavioral nutrition and physical activity* 2013;10(1):135.
34. Baker J, Fraser-Thomas J, Dionigi RA, et al. Sport participation and positive development in older persons. *European review of aging and physical activity* 2010;7(1):3.
35. Eime RM, Harvey JT, Brown WJ, et al. Does sports club participation contribute to health-related quality of life. *Med Sci Sports Exerc* 2010;42(5):1022-8.
36. Oja P, Titze S, Bauman A, et al. Health benefits of cycling: a systematic review. *Scandinavian journal of medicine & science in sports* 2011;21(4):496-509.
37. Finch C. A new framework for research leading to sports injury prevention. *J Sci Med Sport* 2006;9(1):3-9. doi: 10.1016/j.jsams.2006.02.009
38. Filbay SR, Bishop F, Peirce N, et al. Common attributes in retired professional cricketers that may enhance or hinder quality of life after retirement: a qualitative study. *BMJ Open* 2017;7(7):e016541. doi: 10.1136/bmjopen-2017-016541

For peer review only

39. Filbay SR, Bishop FL, Peirce N, et al. Physical activity in former elite cricketers and strategies for promoting physical activity after retirement from cricket: a qualitative study. *BMJ Open* 2017;7(11):e017785. doi: 10.1136/bmjopen-2017-017785
40. Daniel WW. Biostatistics : a Foundation for Analysis in the Health Sciences, Textbook + Student Solutions Manual: John Wiley & Sons Inc 2005.
41. England Rugby. England Rugby's women and girls action plan. - RFU, 2017.
42. Cai H, Bullock GS, Sanchez-Santos MT, et al. Joint pain and osteoarthritis in former recreational and elite cricketers. *BMC Musculoskelet Disord* 2019;20(1):596. doi: 10.1186/s12891-019-2956-7 [published Online First: 2019/12/14]
43. McKay C, Merrett C, Emery C. Predictors of FIFA 11+ implementation intention in female adolescent soccer: An application of the Health Action Process Approach (HAPA) model. *International journal of environmental research and public health* 2016;13(7):657.
44. Perera NKP, Åkerlund I, Hägglund M. Motivation for sports participation, injury prevention expectations, injury risk perceptions and health problems in youth floorball players. *Knee Surgery, Sports Traumatology, Arthroscopy* 2019;27(11):3722-32. doi: 10.1007/s00167-019-05501-7
45. Day MA, Ward LC, Thorn BE, et al. The pain-related cognitive processes questionnaire: development and validation. *Pain Med* 2017;19(2):269-83.
46. Jones M, Davies M, Leyland K, et al. Osteoarthritis and other long-term health conditions in former elite cricketers. *J Sci Med Sport* 2018;21(6):558-63. doi: 10.1016/j.jsams.2017.10.013 [published Online First: 2017/11/02]
47. Clarsen B, Myklebust G, Bahr R. Development and validation of a new method for the registration of overuse injuries in sports injury epidemiology: the Oslo Sports Trauma Research Centre (OSTRC) overuse injury questionnaire. *Br J Sports Med* 2013;47(8):495-502.
48. Clarsen B, Rønsen O, Myklebust G, et al. The Oslo Sports Trauma Research Center questionnaire on health problems: a new approach to prospective monitoring of illness and injury in elite athletes. *Br J Sports Med* 2014;48(9):754-60.
49. Lee PH, Macfarlane DJ, Lam T, et al. Validity of the international physical activity questionnaire short form (IPAQ-SF): A systematic review. *Int J Behav Nutr Phys Act* 2011;8(1):115.
50. Wen J, Yk S, Yp L, et al. Quality of life, physical diseases, and psychological impairment among survivors 3 years after Wenchuan earthquake: a population based survey. *PLoS One* 2012;3
51. European Social Survey European Research Infrastructure Consortium (ESS ERIC). Source Questionnaire [Available from: https://www.europeansocialsurvey.org/methodology/ess_methodology/source_questionnaire/.
52. Diener E, Wirtz D, Tov W, et al. New well-being measures: Short scales to assess flourishing and positive and negative feelings. *Social Indicators Research* 2010;97(2):143-56.
53. NatCen Social Research UCL, Department of Epidemiology and Public Health. Health Survey for England, 2015. [data collection]. UK Data Service, 2017 [Accessed 25 May 2019]. Available from: http://doi.org/10.5255/UKDA-SN-8280-1.
54. Gabbe B, Finch C, Bennell K, et al. How valid is a self reported 12 month sports injury history? *British Journal of Sports Medicine* 2003;37(6):545-47.
55. Jenkins P, Earle-Richardson G, Slingerland DT, et al. Time dependent memory decay. *American Journal of Industrial Medicine* 2002;41(2):98-101. doi: 10.1002/ajim.10035

For peer review only

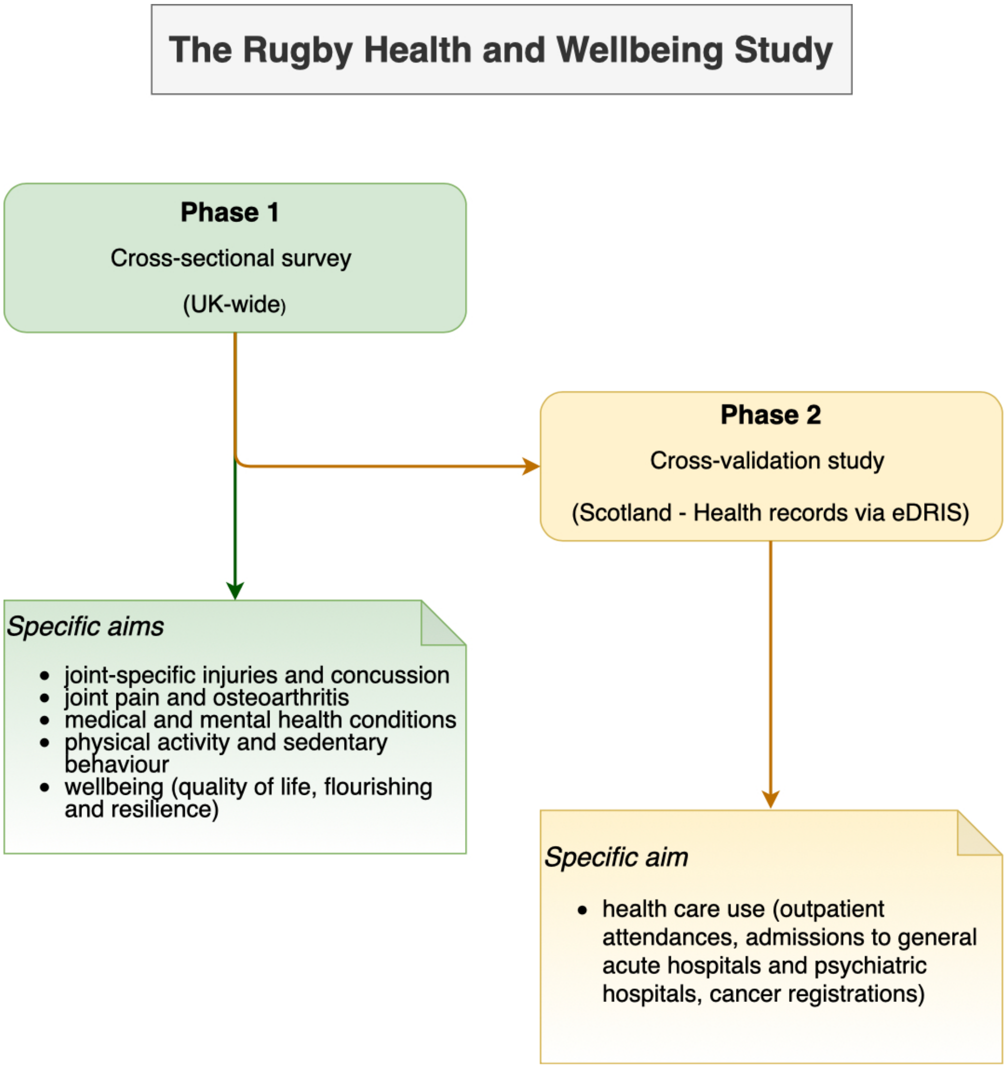

Figure 1 Study design and aims. eDRIS – electronic Data Research and Innovation Service.

90x96mm (300 x 300 DPI)
